# Direct DNA and PNA probe binding to telomeric regions without classical in situ hybridization

**DOI:** 10.1186/1755-8166-6-42

**Published:** 2013-10-08

**Authors:** Matthew D Genet, Ian M Cartwright, Takamitsu A Kato

**Affiliations:** 1 Department of Environmental and Radiological Health Sciences, Colorado State University, 1618 Campus Delivery, Fort Collins, CO 80523, USA

**Keywords:** PNA probes, FISH, Cytogenetics

## Abstract

**Background:**

Fluorescence *in situ* Hybridization (FISH) utilizes peptide nucleic acid (PNA) probes to identify specific DNA sequences. Traditional techniques have required the heat denaturing of the DNA in formamide followed by multiple hours at moderated temperatures to allow the probe to hybridize to its specific target. Over the past 30 years, advancements in both protocols and probes have made FISH a more reliable technique for both biological research and medical diagnostics, additionally the protocol has been shortened to several minutes. These PNA probes were designed to target and hybridize to both DNA and RNA, and PNA-protein interactions still remain unclear.

**Results:**

In this study we have shown that a telomeric single stranded specific PNA probe is able to bind to its target without heat denaturing of the DNA and without formamide. We have also identified a centromere specific probe, which was found to bind its target with only incubation with formamide.

**Conclusions:**

Certain PNA probes are able to hybridize with their targets with minimal to no denaturing of the DNA itself. This limited denaturing preserves the chromosome structure and may lead to more effective and specific staining.

## Background

The PNA probe is similar to a DNA probe, except the phosphate backbone is instead a pseudo-peptide polymer. The pseudo-peptide polymer has no charge, which does not repeal DNA or RNA. This allows the PNA probe to bind to a complimentary sequence of either DNA or RNA with a higher affinity than the DNA or RNA would have when binding with itself [1]. Traditional FISH staining protocols have required factors that denature the DNA double helix in order for the probe to gain sufficient access to the DNA sequence and further hybridize to it [2,3]. This is achieved by exposing the chromosomes to a concentrated solution of formamide at high temperatures, 70 to 80°C, for a few minutes, followed by the addition of the probe which is then allowed to hybridize for multiple hours at 37°C in a formamide solution. Formamide is a useful denaturant as it lowers both the stability and melting point of DNA linearly as the concentration of formamide increases [4]. This hybridization period has been recently shortened to only a few minutes with the use of a microwave; however, the use of formamide in the staining solution was still used [5]. Our study has shown that the telomere PNA and DNA probes were able to bind to the telomeric regions without the use of either heat or formamide, while the centromere PNA probe still require both. It is a well-known fact that cytogenetic analysis, especially analysis of dicentric and centric ring formation, is the most reliable and strongest biomarker for assessing the exposure of an individual who has been exposed to radiation when no physical dose estimate is available. Because of this, it is crucial to understand the full extent of PNA FISH probes [6,7]. Not only is PNA FISH staining effective in identifying dicentrics and other types of chromosomal damages, it also has had implications in CO-FISH and various other assays that address specific gene amplification and deletions [8-11]. We have shown in our study that not only PNA, but also DNA probes have the ability to hybridize with their target, especially telomeric sequences, without denaturing the DNA and minimal incubation. This has the potential to allow for more sensitivity and rapid assays because the DNA is not as altered and retains more of its true structure.

## Results

### Treatment conditions in goat serum, formamide, or traditional denature

**Figure 1:**
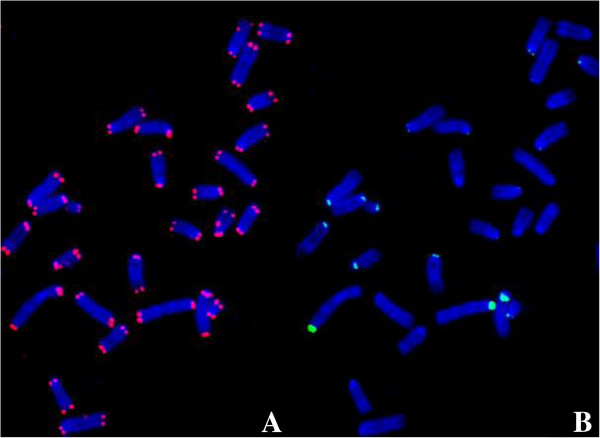
**A metaphase spread of the mouse fibroblast cell line, B70, stained with the TelC-Cy3 and CENPB Box-FAM PNA probes using the direct staining in goat serum without denature protocol with a hybridization of 4 hours at room temperature. A** shows the telomere signal (red), rated as strong while **B** shows the centromere (green), rated as poor.

**Figure 2:**
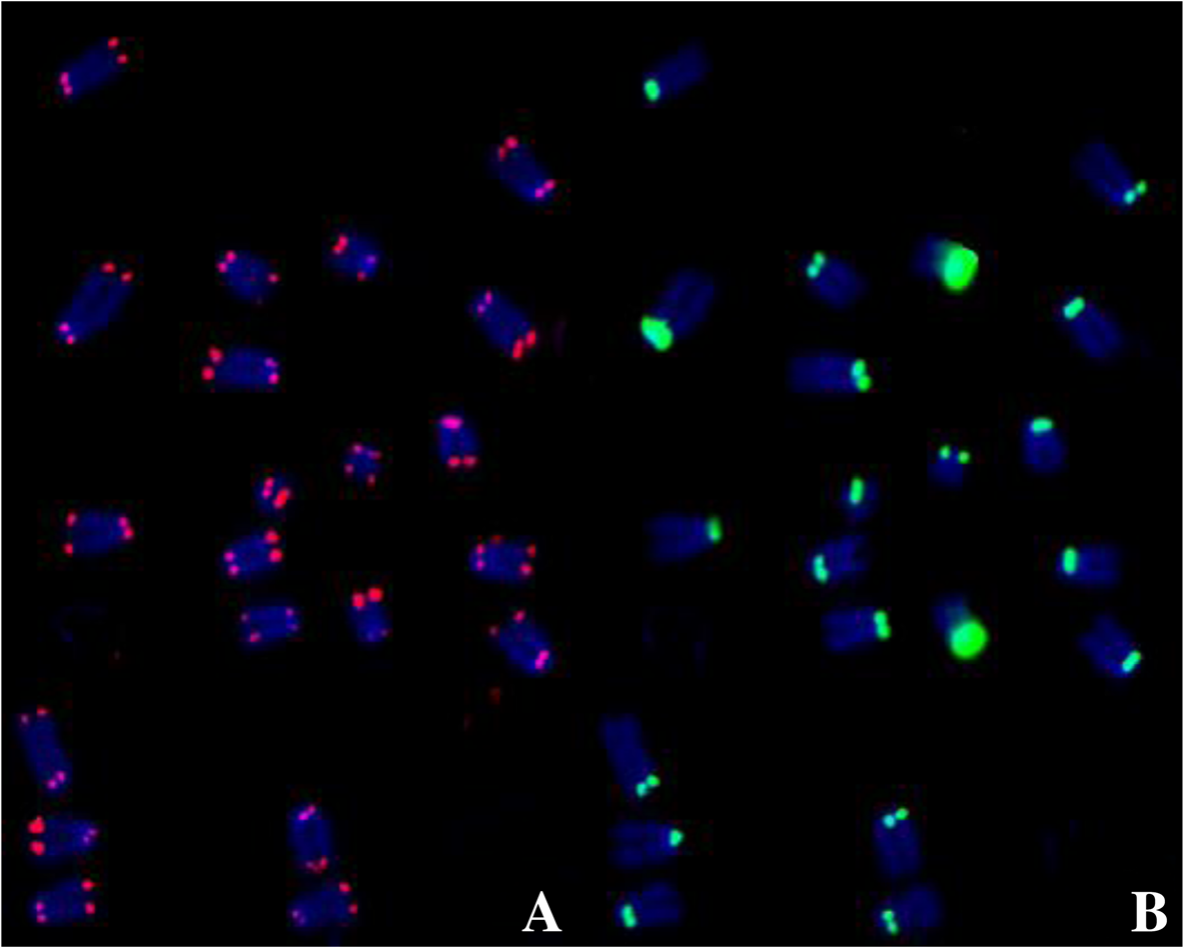
**A metaphase spread allowing with TelC-Cy3 and CENPB Box-FAM PNA probes using the direct staining in formamide without denature protocol with hybridization of 18 hours at room temperature. ** Both the telomere **(A)** and centromere signals **(B)** were rated as strong.

The strength of the signals for TelC-Cy3, TelG-Cy3, CENPB Box-FAM and Cent-FAM in both the FISH protocol and the non-traditional situ hybridization at a hybridization period of 18 hours at 4°C, room temperature, and 37°C were analyzed. In Table 1, the signal strength of the centromere and telomere probes were rated separately as; absent for no staining, poor high background for a signal on only a few chromosomes with high background, fair for over half being stained with moderate signal strength, and strong for a clear signal on all chromosomes. When using the goat serum alone at room temperature only the telomere signals showed on all chromosomes, but some centromere signals were lost in both CENPB Box-FAM and Cent-FAM probes (Figure 1). On the other hand, the formamide without heat denature method, three probes except Cent-FAM probe had a strong signal on all chromosomes (Figure 2). We confirmed all four probes stained on all chromosomes with traditional FISH method. For the non-traditional situ hybridization method, the TelC-Cy3 signal was strong on all chromosomes similar to FISH at all combinations of time periods and temperatures. The TelG-Cy3 signal was present in all cases, however, the signal quality was often rated poor or fair as the signals were not always as strong or present on as many chromosomes as TelC-Cy3. As for the centromeres, CENPB Box-FAM signal was absent at lower temperatures in goat serum, and was only strong in formamide at room temperature or 37°C. Cent-FAM probe signals were absent in both staining solutions at all temperatures.

**Table 1:** The signal strength of the telomere and centromere probes after an 18-hour hybridization period at varying temperatures and conditions were rated as absent, poor, fair or strong

**Probes**	**Goat Serum**			**Formaldehyde**			**FISH**
	**4°C**	**RT**	**37°C**	**4°C**	**RT**	**37°C**	
TelC	Strong	Strong	Strong	Strong	Strong	Strong	Strong
TelG	Poor HB	Fair	Strong	Poor HB	Fair	Strong	Strong
CENPB Box	Absent	Poor	Strong	Fair	Strong	Strong	Strong
Cent	Absent	Absent	Absent	Absent	Absent	Absent	Strong

High background was indicated as, HB, frequently found accompanying that signal only at the specified conditions. FISH indicates the traditional FISH protocol was used.

### Time dependence

The strength of the signals for TelC-Cy3 and CENPB Box-FAM were analyzed at hybridization periods of 1, 4, and 18 hours at room temperature, and rated. Again, TelC-Cy3 showed the strong signals on all chromosomes at all time points. The CENPB Box-FAM signal was absent at a treatment period of 1 hour, poor at 4 hours, and strong at 18 hours.

**Figure 3:**
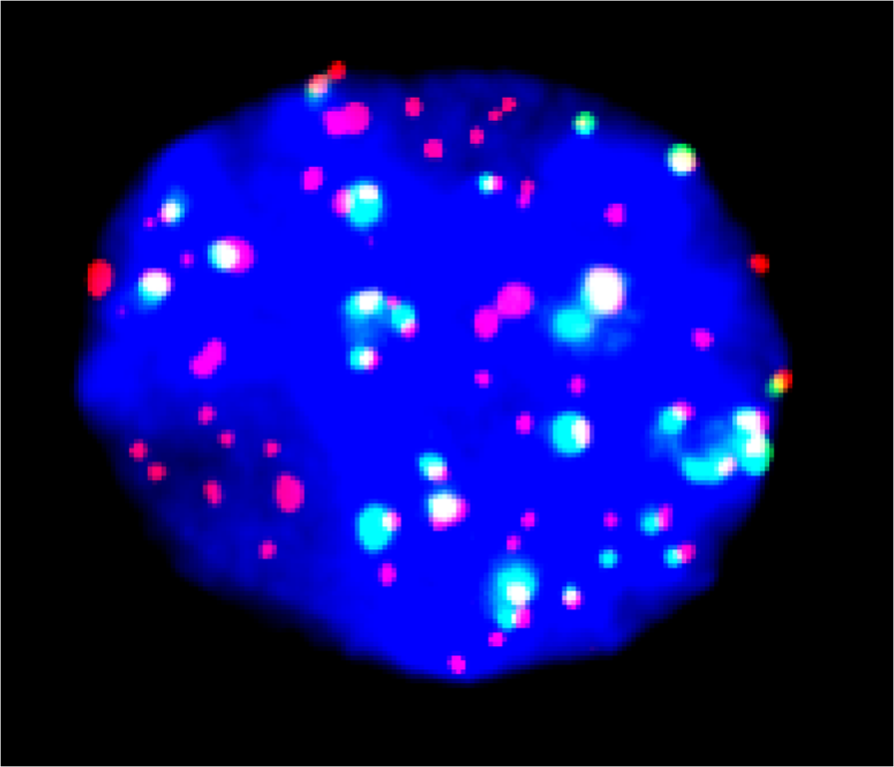
**An interphase nucleus stained with the same conditions as Figure **2**.**

18 hour hybridization at room temperature gives strong signals of TelC-Cy3 and CENPB Box-FAM PNA probes to not only metaphase chromosome spreads, but also interphase nuclei (Figure 3).

### Telomere DNA probes also bind to telomere without heat denature

DNA telomere probes were able to stain the telomere region of all chromosomes when they are incubated room temperature or 37°C, but not 4°C for overnight. Their signal strength was much lower than the PNA counterparts. Without heat denaturing, the PNA probes can achieve fair staining.

## Discussion

In this study, we have shown that the heat-denaturing step in traditional FISH protocols is not required for PNA probes to bind to their target strand of DNA. Additionally, it was also noted that TelC-Cy3 and TelG-Cy3 PNA probes did not require any denaturing to hybridize with their target DNA strand. This supports results from prior studies performed outside the cell and chromosome which indicated that PNA probes have the ability to displace their target double strand DNA and form internal Watson-Crick bonds [12-14].

As discussed in an earlier study, the classic heat denaturing step has been found to not be as critical as once believed for correct binding of DNA probes to their target DNA sequence [15]. The study showed that in an ethylene carbonate buffer DNA probes could hybridize with their target when incubated overnight at 45°C. This supports our findings that PNA probes are capable of displacing a DNA double strand in the presence of only formamide.

Initially, the strong telomere signal may be attributed to the fact that the telomere region of the chromosome is composed of loop structures possibly allowing the PNA probes easier access to single stranded DNA [16]. TelC-Cy3 had a stronger signal than TelG-Cy3 and it is important to note that TelC-Cy3 targets the sequence contained in the single stranded T-Loop. Slides where treated with RNase and Pepsin prior to staining which allows us to rule out the binding of the PNA probe to either single stranded RNA or any proteins associated with the target region of the telomere. The differences in signal strength between telomere and centromere probes can be attributed to their target region and also the target sequence.

In conclusion, this study shows that the need to denature the target DNA with any type of heat, and with certain PNA probes formamide, could possibly be avoided with the use of high affinity PNA probes. These results could possibly lead to more accurate cytogenetic analytics of chromosome aberrations.

## Conclusions

In this study we have effectively demonstrated that telomere and centromere PNA probes could bind to their targets with limited denaturing by formamide alone. It was seen that TelC-Cy3 and TelG-Cy3 PNA probes bound to their telomere target at all temperatures and without formamide treatment. It was noted that CENPB Box-FAM and Cent-FAM did not bind to their target when formamide was omitted from the staining solution, indicating that the target region may play a role in how much the DNA has to be denatured before the PNA probe can bind. These findings suggest that PNA probes have a high enough affinity for their targets that they are able to bind to the target DNA strand without having to have the DNA helix separated.

## Methods

### Cell culturing

B70 mouse fibroblast cell strains were isolated from the skin of female C57/B6 mice, using only early passages 3 and 4. B70 cells were cultured in Minimum Essential Medium Alpha media (Hyclone, ThermoFisher, Waltham, MA) with 15% FBS (Sigma, St. Louis, MO) antibiotics (Anti-Anti, Invitrogen, Indianapolis, IN) [5].

### Chromosome harvesting

Approximately 8 hours before harvesting, 0.1 μg/ml colcemid was added to the flasks to arrest the cells in M-phase. Cells were trypsinized and were suspended in 6 ml of a 75 mM KCl solution warmed to 37°C and placed in a 37°C water bath for 20 min. Carnoy’s solution (3:1 methanol to acetic acid) was added to the samples according to the standard protocol. Slides were placed in ice water and allowed to chill. The cell solution was dropped onto the cold slides. These were set aside and allowed to dry until the Carnoy’s solution had evaporated, roughly 4–5 minutes [7].

### FISH protocol

The slides were first submersed in RNase A (0.1 mg/mL) at 37°C for 10 minutes, followed by a PBS wash. Then, they were placed in 4% paraformaldehyde in PBS for 10 minutes at room temperature, washed in PBS, and then dehydrated in 70%, 85%, and 100% ethanol for two minutes each in an ice water bath. They were then placed in a 2XSSC 70% Formamide solution at 80°C for 2 minutes, followed by the same ethanol wash. The PNA probe solution consisted of 60% of Formamide, 20 mM of Tris–HCl, 200 nM of either TelG-Cy3 (Cy3-O-TTAGGGTTAGGGTTAGGG) or TelC-Cy3 (Cy3-O-CCCTAACCCTAACCCTAA) and 200 nM of either CENPB Box-FAM (FAM-O-ATTCGTTGGAAACGGGA) or Cent-FAM (FAM-O-AAACTAGACAGAAGCATT). This solution was denatured at 85°C for 5 minutes, then cooled down to 37°C before adding 30 μL to each slide. The probes were allowed to hybridize overnight at 37°C, and the slides were then washed in the 2XSSC 70% Formamide solution for 15 minutes at 37°C, followed by 5 minutes in PN Buffer at room temperature. The chromosomes were counter stained with Prolong Gold Antifade with 4′,6-diamidino-2-phenylindole (DAPI) (Invitrogen) [17].

### Direct staining without heat denature protocol

The slides were placed into 4% paraformaldehyde in PBS for 10 minutes at room temperature, and then washed in PBS. Next, the slides were treated in RNase A (0.1 mg/mL) in PBS at 37°C for 15 minutes, followed by pepsin (0.002%) in 100 mM HCl treatment at 37°C for 15 minutes, with washing with PBS in between and after. Finally, the slides were placed in 70%, 85%, and 100% ethanol for two minutes each [5].Then, the probe solution the same as above was used, and in addition, a modified PNA probe solution was also used, consisting of 200 nM of either TelG-Cy3 or TelC-Cy3 and 200 nM of CENPB Box-FAM or Cent-FAM in 60% of Formamide, 20 mM of Tris–HCl, 200 nM or 10% goat serum in PBS. These probe solutions were not heat denatured before adding 30 μL to the slides and secured with a coverslip and allowed to hybridize at either 4°C, room temperature, or 37°C for either 1, 4, or 18 hours. After hybridization, the slides were submersed in 1X PN buffer for 5 minutes at 37°C, followed by a 5-minute wash in PBS at room temperature. The chromosomes were counter stained with Prolong Gold Antifade with 4′,6-diamidino-2-phenylindole (DAPI) (Invitrogen). Direct staining without heat denature with DNA TelC-Cy3 and TelG-Cy3 probes were carried out in the same manner as a PNA probe. Those DNA telomere probes have same sequence as their PNA counter parts.

### Fluorescence imaging

A Zeiss Axioplan fluorescence microscope (Zeiss, Oberkochen, Germany) was used with a Q-imaging Aqua cooled CCD camera (Q-imaging, Surrey, BC, Canada). Images were combined using QCapture Pro 6.0 software.

## Competing interest

The authors declare that they have no competing interest.

## Authors’ contributions

MG contributed primarily to image analysis and writing of the manuscript. IC contributed to the experiments and the writing of the manuscript. TK oversaw the project in its entirety. All authors read and approved the final manuscript.
